# Neonatal Submandibular Sialadenitis Without Abscess Formation: A Rare Cause of Neck Swelling

**DOI:** 10.7759/cureus.106526

**Published:** 2026-04-06

**Authors:** Sania Shahid, Jarisha Ali, Alaa W Qanbar, Rania Eladl, Sana Ahmed, Aqsa Shahid

**Affiliations:** 1 Pediatrics, Al Jalila Children's Specialty Hospital, Dubai, ARE; 2 Medicine, Dubai Medical University, College of Medicine, Dubai, ARE; 3 Medicine, University of Georgia, Tbilisi, GEO

**Keywords:** emergency, pediatrics, pediatrics emergency, sialadenitis, swelling of neck, term neonate

## Abstract

Acute neonatal sialadenitis is a rare condition, most commonly involving the parotid gland, whereas isolated submandibular gland involvement is uncommon and may pose diagnostic challenges. We report the case of a two-week-old term male infant who presented with right submandibular swelling in the absence of fever, systemic signs of infection, or abscess formation. Laboratory evaluation revealed leukocytosis with elevated inflammatory markers, and ultrasound imaging confirmed acute submandibular sialadenitis. The infant responded well to intravenous antibiotics without the need for surgical intervention. This case highlights the clinical variability of neonatal sialadenitis and emphasizes the importance of early recognition, appropriate imaging, and targeted antimicrobial therapy to prevent complications.

## Introduction

Neonatal sialadenitis is a rare inflammatory condition of the salivary glands, with the parotid gland being most frequently affected due to its serous secretion and anatomical susceptibility to ascending infections [1-3]. Submandibular sialadenitis in neonates is significantly less common and has been sparsely reported in the literature [1,4]. Risk factors include dehydration, prematurity, ductal obstruction, stasis of secretions, and bacterial colonization [1,2].

Clinical presentation may range from localized glandular swelling to systemic sepsis if not promptly treated [1,2]. The diagnosis is supported by imaging, typically ultrasonography, which aids in differentiating cellulitis, abscess formation, duct obstruction, and sialolithiasis [4,5]. Early initiation of antibiotics is crucial to prevent complications such as abscess formation, airway compromise, or extension into deep neck spaces [1-3].

We present a case of isolated right submandibular sialadenitis in a two-week-old term neonate with no identifiable risk factors, managed successfully with intravenous antibiotics to avoid adverse outcomes [1,4]

## Case presentation

A male infant born at 37 weeks of gestation via spontaneous vaginal delivery, weighing 2.9 kg, had an antenatal course notable for polyhydramnios and bilateral pleural effusions more pronounced on the right side. Postnatal evaluation showed normal respiratory findings, and chest imaging was unremarkable. The newborn was admitted briefly to the neonatal intensive care unit (NICU) for observation. A sepsis screen was performed, and he received three days of empiric antibiotics before being discharged well. Feeding, weight gain, and general activity remained appropriate over the first two weeks of life.

On day 14 of life, the parents reported a newly noticed swelling beneath the infant’s right jaw. On examination, a well-defined, firm, non-fluctuant swelling measuring approximately 1 cm was present in the right submandibular region. The overlying skin was intact and without erythema, warmth, or discharge. The infant remained afebrile, alert, and feeding normally and exhibited no respiratory distress, irritability, vomiting, or lethargy. Vital signs and systemic examination were stable.

Given the presence of submandibular swelling, a partial neonatal sepsis screen was performed. An initial ultrasound of the neck suggested submandibular sialadenitis. Empirical intravenous antibiotics with ampicillin and cefotaxime were initiated as per the American Academy of Pediatrics (AAP) neonatal sepsis guidelines [6]. Following consultation with the infectious diseases team, ampicillin was discontinued, and cefotaxime monotherapy was continued for a total duration of seven days, with good clinical response.

Laboratory evaluation revealed a white blood cell (WBC) count of 24.6 × 10³/µL (reference range: 7.0-23.0 × 10³/µL), demonstrating marked leukocytosis with neutrophilic predominance and mildly elevated C-reactive protein (CRP) 7.5 mg/L (reference range: 0.6-6.1 mg/L) and procalcitonin 0.23 ng/mL (reference range: <0.05 ng/mL). A basic metabolic panel showed mild hyperkalemia (6.5 mmol/L) and borderline elevated serum calcium (11.2 mg/dL), with all remaining electrolytes and renal function parameters within normal limits (Table 1). The initial electrolyte abnormalities, including hyperkalemia and borderline hypercalcemia, were likely attributable to sampling-related factors, as repeat measurements were within normal limits, suggesting pseudohyperkalemia rather than true metabolic derangement.

**Table 1:** Laboratory investigations on admission.

Laboratory Parameter	Result	Reference Range
Inflammatory Markers		
C-reactive protein (CRP)	7.5 mg/L (High)	0.6-6.1 mg/L
Procalcitonin	0.23 ng/mL (High)	<0.05 ng/mL
Complete Blood Count		
White blood cell count	24.6 ×10³/µL (High)	7.0-23.0 ×10³/µL
Neutrophils (absolute)	15.25 ×10³/µL (High)	3.0-5.0 ×10³/µL
Hemoglobin	14.2 g/dL (Low)	15.0-21.0 g/dL
Mean corpuscular volume (MCV)	75.0 fL (Low)	92.0-118.0 fL
Platelet count	586 ×10³/µL (High)	210-500 ×10³/µL
Basic Metabolic Panel		
Sodium	134 mmol/L	133-146 mmol/L
Potassium	6.5 mmol/L (High)	4.2-6.0 mmol/L
Creatinine	0.41 mg/dL	0.4-1.0 mg/dL
Total calcium	11.2 mg/dL	8.6-11.0 mg/dL

Blood cultures obtained prior to antibiotic administration showed no growth after five days. Peripheral blood film demonstrated severe absolute neutrophilic leukocytosis with toxic granulation, reactive lymphocytes, microcytic hypochromic red cells, and mild thrombocytosis, consistent with an inflammatory or infectious process.

A repeat complete blood count performed on day five of treatment showed an improvement in leukocytosis, with WBC decreasing to 12.0 × 10³/µL (reference: 7.0-23.0 × 10³/µL), and normalization of inflammatory markers.

**Figure 1:**
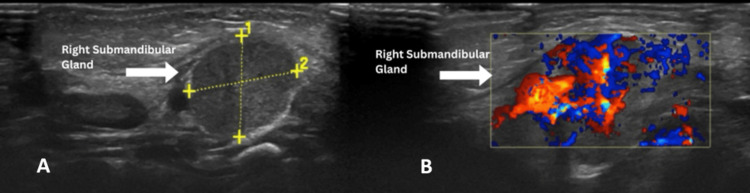
Ultrasound of the right submandibular gland. A: a mildly bulky right submandibular gland measuring 1.2 × 1.1 cm, hypoechoic with evidence of mild capsular thickening and adjacent fat stranding (arrow), no evidence of duct dilatation and calcification; B: increased vascularity and pericapsular inflammation (arrow).

**Figure 2:**
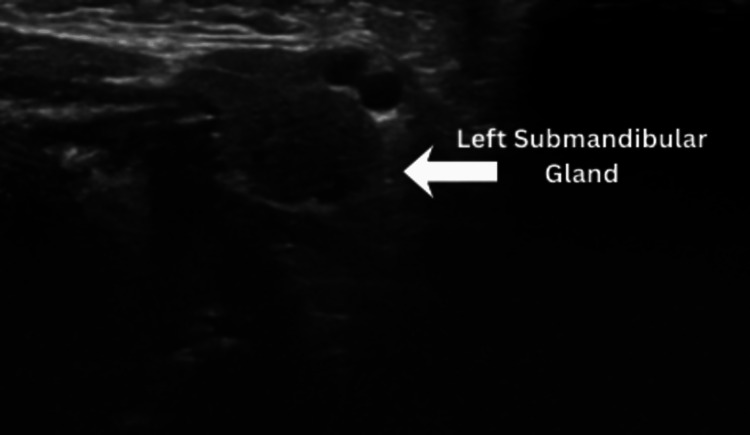
Comparison with the normal left submandibular gland (arrow).

An initial neck ultrasound demonstrated mild enlargement of the right submandibular gland measuring 1.2 × 1.1 cm, with hypoechoic echotexture, increased vascularity, and periglandular fat stranding, suggestive of glandular inflammation (Figure 1). A small right jugulodigastric lymph node measuring 0.47 cm was noted adjacent to the gland, likely reactive in nature and secondary to the associated glandular inflammation. The contralateral submandibular (Figure 2) and parotid glands appeared normal. No ductal dilatation, sialolithiasis, liquefaction, or abscess formation was identified. Image quality was limited by the frequency and size of the portable linear probe; however, findings were sufficient to support a diagnosis of acute right submandibular sialadenitis.

The differential diagnosis for submandibular swelling in a neonate is broad and includes both congenital and acquired conditions. Congenital lesions such as branchial cleft cysts, thyroglossal duct cysts, and lymphatic malformations may present as neck swellings during early infancy. Other considerations include reactive lymphadenitis, salivary duct obstruction, sternocleidomastoid tumor of infancy, and infectious sialadenitis.

In this case, given the presence of localized glandular enlargement, elevated inflammatory markers, and supportive ultrasound findings, an infective etiology, specifically acute submandibular sialadenitis, was favored over congenital or obstructive causes. The absence of fever and systemic compromise did not exclude infection, as neonatal glandular infections may present with minimal constitutional symptoms.

The infant received intravenous ampicillin and cefotaxime on admission. Ampicillin was discontinued after negative cultures; cefotaxime was continued for a total of seven days as per infectious disease recommendations. The swelling regressed progressively during the observation period.

At discharge, the infant was afebrile and remained clinically stable throughout the hospital course. He was reviewed in the infectious disease clinic two days later and continued to do well.

## Discussion

Neonatal sialadenitis is a rare inflammatory condition of the salivary glands, occurring most commonly in the parotid gland [1]. Submandibular gland involvement is significantly less common than parotid involvement in neonatal sialadenitis due to anatomical and physiological differences, with only a few isolated cases described in the literature. The parotid gland has a predominantly serous secretion with lower antimicrobial properties and a longer duct (Stensen’s duct), which may predispose to stasis and ascending infection. In contrast, the submandibular gland produces mixed (serous and mucous) saliva with relatively higher antimicrobial activity and has a shorter, wider duct (Wharton’s duct), which may reduce the risk of infection. These factors likely contribute to the lower incidence of submandibular sialadenitis compared to parotitis [1]. Its rarity may lead to diagnostic delays, especially in asymptomatic or minimally symptomatic infants [2].

Risk factors for neonatal salivary gland infections include dehydration, low birth weight, prolonged orogastric feeding, ductal obstruction, and immunologic immaturity [3]. However, our patient was a healthy term neonate with no identifiable risk factors, a pattern occasionally reported in isolated cases [4]. The absence of systemic symptoms further contributed to the diagnostic challenge. In this context, differential diagnoses for unilateral submandibular swelling include lymphadenitis, cellulitis, congenital lesions such as branchial cleft cysts, abscess formation, and, less commonly, vascular or neoplastic causes.

Ultrasonography is the first-line imaging modality due to its safety, rapid availability, and ability to distinguish inflammatory changes from congenital neck masses [5]. In this case, ultrasonography demonstrated hallmark features of sialadenitis, including glandular enlargement, increased vascularity, and fat stranding, while also ruling out abscess formation or sialolithiasis. These findings are consistent with previous reports of neonatal submandibular sialadenitis [7]. CT imaging was not undertaken in this case due to the absence of a clinical indication. Ultrasound was sufficient for diagnosis, and considering the risks of radiation exposure in neonates, CT is typically reserved for cases with diagnostic uncertainty or suspected complications.

Early initiation of broad-spectrum intravenous antibiotics is essential to prevent complications such as abscess formation, airway compromise, or deep neck space involvement [5]. Empirical antibiotic therapy was initiated in accordance with established neonatal sepsis guidelines, including those of the AAP, and aligned with our institutional protocol. The combination of ampicillin and cefotaxime provided broad-spectrum coverage against common neonatal pathogens, including Group B Streptococcus, *Listeria monocytogenes*, and Gram-negative organisms such as *Escherichia coli* [3]. Although *Staphylococcus aureus* is a recognized cause of neonatal sialadenitis [3], the absence of clinical features suggestive of staphylococcal infection, such as abscess formation or purulent discharge, and a clinically well child, did not necessitate targeted anti-staphylococcal therapy. In the absence of clinical or microbiological evidence of *Listeria monocytogenes* or enterococcal infection, and with evolving features consistent with localized infection of the gland, antibiotic therapy was rationalized. Ampicillin was discontinued to minimize unnecessary broad-spectrum exposure, while cefotaxime was continued to provide adequate coverage for likely pathogens and ensure effective tissue penetration. Negative blood cultures do not exclude bacterial sialadenitis, as culture-negative cases have been documented, particularly when antibiotic therapy is initiated prior to sampling [8]. 

Our patient demonstrated rapid clinical improvement with cefotaxime therapy alone, similar to outcomes reported in prior cases where non-surgical management was successful [4,8]. The referenced adult case is included to highlight a comparable non-surgical management strategy while acknowledging the limitations in directly extrapolating findings to the neonatal population.

This case reinforces the importance of maintaining a high index of suspicion for sialadenitis in neonates presenting with isolated submandibular swelling and highlights the diagnostic value of ultrasonography, even in the absence of systemic symptoms.

## Conclusions

Neonatal submandibular sialadenitis is a rare but clinically important condition presenting with glandular swelling and elevated inflammatory markers. Diagnosis relies heavily on ultrasonography to differentiate sialadenitis from congenital or obstructive causes of neck swelling. Early recognition and timely initiation of antibiotics can prevent complications and allow for complete resolution. Even healthy term infants may develop sialadenitis, highlighting the need for vigilance when evaluating neonatal neck swellings. 

This case is clinically significant as it demonstrates a rare case of neonatal submandibular sialadenitis in the absence of identifiable risk factors, which may lead to diagnostic uncertainty, particularly in the setting of limited published data. Such presentations can pose challenges for clinicians and contribute to parental anxiety. Reporting cases with varying clinical features and management approaches can aid in improving recognition and guiding appropriate management; in our case, available literature provided reassurance that the chosen approach was appropriate. It also underscores the importance of early recognition and supports effective non-surgical management, contributing valuable insight to the limited existing literature.
